# Landfills and preterm birth in the Guadeloupe archipelago (French West Indies): a spatial cluster analysis

**DOI:** 10.1186/s41182-018-0130-9

**Published:** 2019-01-09

**Authors:** Marion Istvan, Florence Rouget, Léah Michineau, Christine Monfort, Luc Multigner, Jean-François Viel

**Affiliations:** 1 Univ Rennes, CHU Rennes, Inserm, EHESP, Irset (Institut de recherche en santé, environnement et travail) - UMR_S 1085, 2 rue Henri Le Guilloux, F-35000 Rennes, France; 2 Univ Rennes, Inserm, EHESP, Irset (Institut de recherche en santé, environnement et travail) - UMR_S 1085, F-97000 Pointe-à-Pitre, France; 3 Univ Rennes, Inserm, EHESP, Irset (Institut de recherche en santé, environnement et travail) - UMR_S 1085, F-35000 Rennes, France

**Keywords:** Preterm birth, Environmental exposure, Landfill, Spatial clustering, French West Indies

## Abstract

**Background:**

A high rate of preterm birth is observed in the Guadeloupe archipelago (French West Indies), raising the hypothesis of harmful environmental exposures, including landfilling. Our aim was to evaluate whether preterm births cluster around the three main open landfills located in this area.

**Methods:**

The study population consisted of 911 women enrolled in the Timoun mother-child cohort (2004–2007). Home addresses during pregnancy and locations of landfills were geocoded. To test for the presence of preterm birth clusters around each dumpsite, we used a focused cluster test specifically designed to detect spatial clustering around point sources.

**Results:**

A total of 144 (15.8%) preterm births were observed among 911 births. Using the term births (*n* = 767) as controls, a significant cluster was identified within 2 km around the Saint-François landfill with a relative risk (RR) of 4.82 (*p* = 0.04). No clusters were found around the other two landfills (RR = 2.01, *p* = 0.26 and RR = 1.06, *p* = 0.64, for La Gabarre and Baillif, respectively).

**Conclusion:**

The paucity of data available on open landfill sites regarding waste quantities, composition, and changes over time precludes any site-specific interpretation because of the variable degree of possible emissions. This result has to be confirmed in other tropical island environments where waste management has become a major concern with the potential to negatively impact the environment and public health.

## Background

A high rate of preterm birth is observed in the Guadeloupe archipelago (part of the French West Indies): 15.8% (2005–2008) versus 5.5% in mainland France (2010) [1, 2]. This high preterm birth rate cannot be fully explained by the African origin of its population, leaving room for unknown or unstudied risk factors, including environmental exposures.

Waste management has always been a crucial issue in island environments due to limited land space and increased volumes and diversity of solid waste (due to rapid population growth, heavy reliance on imported goods, lack of recycling initiatives, or poor waste collection systems). In this context, landfilling is the major (if not the only) option for waste management. However, landfills can represent a health risk for residents because of exposure to pollutants through different pathways: inhalation of substances emitted by the site, contact with water or polluted soil, and consumption of contaminated foodstuffs or drinking water. In this respect, residential proximity to landfills has been previously linked to preterm births or congenital malformations, although several alternative explanations (including ascertainment bias and residual confounding) cannot be excluded [3, 4].

Spatial epidemiology can give important clues when searching for putative environmental causes of a disease [5, 6]. In particular, cluster analysis allows researchers to assess whether people with the disease have lived closer to one another than would be expected by chance during a specific time period. Local clustering methods can be differentiated as either non-focused or focused tests. Non-focused methods aim at detecting clusters without a priori knowledge of their location. Subsequently, hypotheses about exposures potentially responsible for such clusters can be made. Focused cluster tests are used when there is a priori knowledge about the location of hypothesized clusters (such as suspected or known sources of environmental contaminants) assuming that disease clusters follow the spatial patterns of environmental contaminant dispersion [7].

Because three main open landfills were used for solid waste disposal in the Guadeloupe archipelago, our aim was to evaluate whether preterm births cluster around these predefined locations.

## Methods

Guadeloupe is an archipelago located in the Leeward Islands in the Caribbean. It covers an area of 1628 km^2^ with a population of 450,000 inhabitants. This study relies on the Timoun mother-child cohort fully described elsewhere [1]. Between 2004 and 2007, 1068 women residing in Guadeloupe for more than 3 years were enrolled during the third trimester of pregnancy. In the current study, we excluded women not born in the Caribbean (*n* = 110) to reduce heterogeneity as African ancestry is a strong risk factor for preterm birth [8], cases involving multiple births (*n* = 25), severe birth defects (*n* = 8), and induced pregnancies after fertility treatment (*n* = 15), resulting in a study population of 911 women (one case involved both fertility treatment and multiple births). Gestational age in weeks was estimated by the obstetricians in charge of follow-ups. It was based on the first day of the last menstrual period and was confirmed or corrected by ultrasound. Preterm birth was defined as a birth before 37 completed weeks of gestational age.

**Fig. 1:**
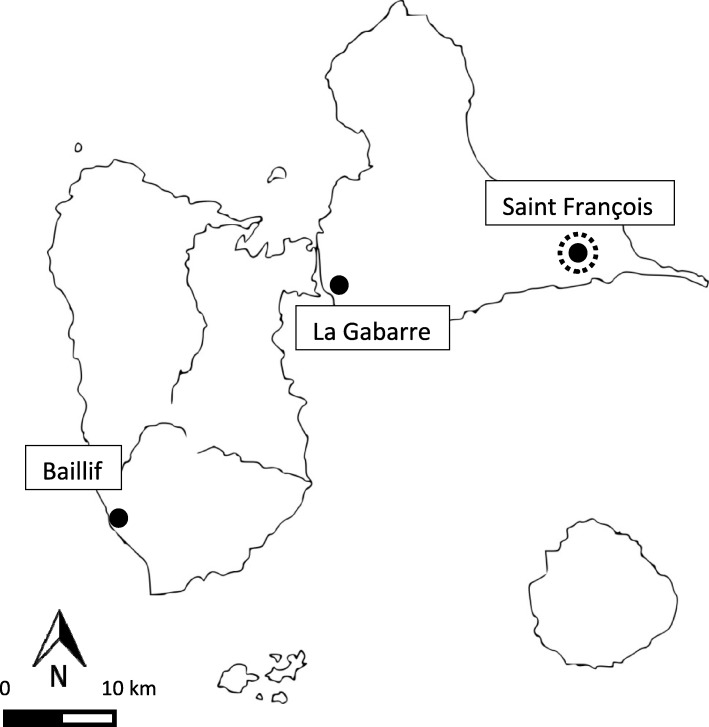
Map of Guadeloupe archipelago (French West Indies), with locations of the three main open landfills (the dotted circle indicates a significant cluster within a 2-km radius of the landfill)

The three main (and official) open landfills in Guadeloupe (namely, La Gabarre, Saint-François, and Baillif) opened as temporary structures in the 1970s (Table 1, Fig. 1). They then received increasing amounts of both municipal waste and hazardous waste (scrap cars, chemical waste, hospital waste, metal waste, industrial waste) in an uncontrolled and uncovered manner [9].

**Table 1:** Description of the three main open landfills in the Guadeloupe archipelago

	La Gabarre	Saint-François	Baillif
Geographic coordinates	16.25956 N 61.54151 W	16.27477 N 61.28276 W	16.03139 N 61.74791 W
Municipality	Les Abymes	Saint François	Baillif
Operation	Opened in 1973 Rehabilitation in 2009	Opened in 1974 Closed in 2010 Rehabilitation in 2012	Opened in 1974 Closed in 2008 Rehabilitation in 2008
Acreage	37 ha	8.6 ha	7 ha

Home addresses during pregnancy and locations of dumpsites were geocoded. To test for the presence of preterm birth clusters around each landfill, we used a (purely spatial) focused cluster test specifically designed to detect spatial clustering around point sources, assuming a Bernoulli distribution and using a grid file with only a single grid point reflecting the coordinates of the focus of interest [10]. The program scans for clusters of geographic size between zero and some upper limit. As a compromise between the need for spatial precision and the limited data available on landfill sites, the maximum radius was set as 2 km around each site, at the likely limit of dispersion for landfill emissions to include both air and water pathways according to a WHO report [11].

For hypothesis testing, we generated 9999 Monte Carlo replications to ensure good statistical power. Focused cluster tests were performed around each landfill in turn using the SaTScan software program (Kulldorff M., Boston, and Information Management Services, Inc., Calverton, Maryland) [12].

## Results

In the study population (*n* = 911), a total of 144 (15.8%) preterm births were observed in as many different locations. Selected socio-demographic and lifestyle characteristics of the study population are reported in Table 2. No significant differences were found between mothers of preterm infants and mothers of full-term infants.

**Table 2:** Socio-demographic characteristics of the study population according to the pregnancy outcome (Timoun cohort, *n* = 911, Guadeloupe archipelago, 2004–2007) (from Rouget et al. 2013)

	Term births *n* = 767	Preterm births *n* = 144	HR (95% CI)
Maternal place of birth			
Guadeloupe or Martinique	669	128	Reference
Other Caribbean islands	98	16	0.9 (0.5–1.5)
Maternal age (years)			
< 20	63	11	1.0 (0.5–1.9)
20–34	437	80	Reference
≥ 35	247	53	1.3 (0.9–1.9)
Years of education			
< 5	49	13	1.7 (0.9–3.0)
5–12	564	101	Reference
> 12	154	30	1.1 (0.7–1.6)
Mother’s employment during pregnancy			
No	449	83	Reference
Yes	318	61	1.0 (0.7–1.4)
Tobacco smoking during pregnancy			
No	737	138	Reference
Yes	30	6	0.9 (0.4–2.2)
Alcohol drinking during pregnancy			
No	745	141	Reference
Yes	22	3	0.8 (0.2–2.4)

*HR* hazard ratio, *CI* confidence interval

Using the term births (*n* = 767) as controls, a significant cluster was identified within 2 km around the Saint-François landfill with a relative risk (RR) of 4.82 (*p* = 0.04) (Table 3). The number of observed preterm births was twice the expected number around the La Gabarre dumpsite (RR = 2.01), yet this difference was not statistically significant (*p* = 0.26). No risk was highlighted in the vicinity of the Baillif landfill (*p* = 0.64).

**Table 3:** Clusters of preterm births in the vicinity of open landfills (Timoun cohort, Guadeloupe archipelago, 2004–2007, 2-km radius, focused tests)

	La Gabarre	Saint-François	Baillif
Timoun newborns	16	4	18
Observed cases	5	3	3
Expected cases	2.53	0.63	2.85
Relative risk	2.01	4.82	1.06
*P* value	0.26	0.04	0.64

## Discussion

We found a significant cluster of preterm birth in the vicinity of the Saint-François landfill, which was located in a less densely populated area than the other two landfills (as reflected by the numbers of Timoun newborns and expected cases). Potential socio-demographic confounding factors did not appear to account for this observation.

The present study has several strengths. First, because of the absence of heavy industries in the Guadeloupe archipelago, no confounding by their effluents is to be predicted. Moreover, no other pollution sources are documented in the areas that surround the three dumpsites. Second, preterm birth was carefully and uniformly characterized within a cohort framework. Both the residential addresses of the pregnant women and the landfill locations were known with sufficient precision to allow appropriate geocoding. Third, this study benefited from the well-established advantages of the focused cluster test to assess whether cases are closer to the sources than expected. Addressing a specific hypothesis of concern, this inferential method has a higher power compared to other spatial methods [13].

This study is not without limitations. First, only authorized landfills were considered; however, illegal, uncontrolled landfills that receive waste without any selection at the origin may be an additional health concern. Second, the distance from the dumpsite was used as a proxy for exposure as it may reflect and integrate a broad range of contaminants and different routes of exposure (not only air but also the contamination from soil or groundwater in the vicinity of the landfill). This buffer-based approach considers that emissions from a landfill are uniformly dispersed in all directions. It is worth noting that a 2-km radius, which is within plausible estimates of the range of chemicals dispersed from a site, has been often used to estimate exposure [13]. However, assuming that environmental exposure is equally distributed around the polluted site might be questionable for specific pollutants. Third, because of the limited number of preterm births, the possibility of insufficient statistical power to indicate clusters around the Gabarre dumpsite cannot be entirely dismissed. Unfortunately, no national birth register is available in France, and we had to rely upon a locally run mother-child cohort. Fourth, a limitation inherent to any landfill is the wide variety of pollutants, exposure pathways and exposure scenarios, entailing a large complexity and difficulty in estimating the health risks possibly involved in its vicinity. Unfortunately, specific characteristics of the dumpsites (e.g., age, waste streams disposed of, waste quantities, operating practices, changes over time, conditions at the bottom layer) are generally not known and relevant data sets are simply not available. In this regard, further elaborating on the apparently discrepant results (the significant cluster is not observed in the vicinity of the largest landfill, i.e., La Gabarre) would be unwise.

To the best of our knowledge, the current study is the first to report on this topic in a tropical island environment. The available scientific evidence has been recently reviewed by Kihal-Talantikite et al. [13], revealing scarce literature for preterm birth. An increased risk was found with residential proximity to a hazardous waste landfill in New Jersey (USA) (OR [odds ratio] = 2.10; 95% CI [confidence interval] 1.01–4.36) [14] and to a hazardous waste site in Nova Scotia (Canada) (RR [relative risk] = 1.13; 95% CI 1.04–1.22) [15]. Conversely, no association was found in the vicinity of a municipal solid waste landfill in Quebec (Canada) (OR = 1.00; 95% CI 0.88–1.13) [16] or around open dumpsites in Alaska (USA) (OR = 1.09; 95% CI 0.78–1.51) [17]. One must, however, keep in mind that the complex nature of the landfill ecosystem makes it difficult to compare studies and draw lessons from other locations.

## Conclusion

Waste management in island environments has become a major concern with the potential to negatively impact development activities (including tourism and trade), food supplies, the environment, and public health. The excess risk of preterm birth observed around one Guadeloupian dumpsite supports these concerns but will need to be confirmed in other tropical island environments. In the meantime, preterm births might be considered in any risk assessment of landfill disposal sites in tropical belt.

## Abbreviations

OR: Odds ratio
RR: Relative risk
